# Intra-abdominal fat is related to metabolic syndrome and non-alcoholic fat liver disease in obese youth

**DOI:** 10.1186/1471-2431-13-115

**Published:** 2013-08-07

**Authors:** Loreana Sanches Silveira, Paula Alves Monteiro, Bárbara de Moura Mello Antunes, Patrícia Monteiro Seraphim, Rômulo Araújo Fernandes, Diego G Destro Christofaro, Ismael F Freitas Júnior

**Affiliations:** 1 Department of Physiotherapy, UNESP, 421 Garcia Paes St. Jardim Paulista, 19023-060 Presidente Prudente, SP, Brazil; 2 Department of Physical Education, UNESP, Rio Claro, SP, Brazil; 3 Department of Physical Education, UNESP, Presidente Prudente, SP, Brazil

**Keywords:** Obesity, Children, Adolescents, Metabolic syndrome, Non-alcoholic fatty liver disease, Trunk fat

## Abstract

**Background:**

Previous studies have shown an association between adiposity, especially intra-abdominal adipose tissue, and hemodynamic/metabolic comorbidities in adults, however it is not clear in pediatric population. The aim of the study was to analyze the relationship between non-alcoholic fatty liver disease (NAFLD) and components of metabolic syndrome (MS) with values of intra-abdominal (IAAT) and subcutaneous (SCAT) adipose tissue in obese children and adolescents.

**Methods:**

Cross-sectional study. Subjects: 182 obese sedentary children and adolescents (aged 6 to 16 y), identified by the body mass index (BMI). Measurements: Body composition and trunk fat by dual-energy X-ray absorptiometry- DXA; lipid profile, blood pressure and pubertal stage were also assessed. NAFLD was classified as absent (0), mild (1), moderate (2) and severe (3), and intra-abdominal and subcutaneous abdominal fat thickness were identified by ultrasound. The MS was identified according to the cut offs proposed by World Health Organization adapted for children and adolescents. The chi-square test was used to compare categorical variables, and the binary logistic regression indicated the magnitude of the associations adjusted by potential cofounders (sex, age, maturation, NAFLD and HOMA-IR).

**Results:**

Higher quartile of SCAT was associated with elevated blood pressure (p = 0.015), but not associated with NAFLD (p = 0.665). Higher IAAT was positively associated with increased dyslipidemia (p = 0.001), MS (p = 0.013) and NAFLD (p = 0.005). Intermediate (p = 0.007) and highest (p = 0.001) quartile of IAAT were also associated with dyslipidemia, independently of age, sex, maturation, NAFLD and HOMA-IR (homeostatic model assessment-insulin resistance).

**Conclusion:**

Obese children and adolescents, with higher IAAT are more prone to develop MS and NAFLD than those with higher values of SCAT, independent of possible confounding variables.

## Background

Changes observed in modern society in the last decades have led to increasing prevalence of childhood obesity in developed and developing countries [1]. Pediatric obesity is associated with a large variety of metabolic/hemodynamic comorbidities, such as the metabolic syndrome (MS) and non-alcoholic fat liver disease (NAFLD) [2,3].

Deeper analysis have shown that, not only the total, but the distribution of excessive adipose tissue seems to determine the development of these comorbidities and, therefore, abdominal obesity (intra-abdominal adipose tissue [IAAT]) is point out as more harmful to human health than subcutaneous adipose tissue (SCAT) [4,5].

It is well documented that adulthood health is affected by risk factors which may determine cardiovascular diseases, such as dyslipidaemia, diabetes type II and hypertension at early age [6-8] and is fundamental to detect these potential risk factors to prevent or treat obesity. On the other hand, it is not clear whether the above mentioned influence of body fat distribution occur among obese children and adolescents, due to the lack of research about the relationship between IAAT and SCAT with MS and NAFLD in these populations.

The purpose of the present study is to analyze the relationship of NAFLD and components of MS with values of IAAT and SCAT, in a sample of sedentary obese children and adolescents.

## Methods

### Participants and setting

The present study included 182 children and adolescents of both genders (51.6% females), who met the inclusion criteria: (a) obesity identified by body mass index (BMI) according to Cole *et al*. (2000) [9], (b) aged between 6 and 16 years (at evaluation date) and (c) no regular physical activity practice in the last three months prior to the assessment and no disease or limitation that does not allowed the practice (d) sign a written informed consent form for the participation in the study (participants and parents/guardians).

The number of participants at each age ranged from 20 (6 and 7 years), 44 (8 and 9 years), 51 (10 to 12 years) 48 (13 and 14 years) and 19 (15 and 16 years).

The ethical committee of the FCT/UNESP approved this study (Protocol number 07/2009).

### Anthropometry

Body weight (BW) was measured with an electronic scale (precision 0.1 kg [Filizzola PL 150, Filizzola® Ltda]) and the height with a wall-mounted stadiometer (precision 0.1 cm [Sanny®, São Paulo, Brazil]), with the subjects wearing light clothing and no shoes. Anthropometric measurements were performed by trained researchers, according to standardized techniques [10].

### Dual energy X-ray absorptiometry

Percentage of body fat (%BF) and amount of trunk fat mass (TFM [kg]) were estimated by a Dual-energy X-ray absorptiometry- DXA scanner (Lunar DPX-NT; General Electric Healthcare, Little Chalfont, Buckinghamshire [software version 4.7]). The measurement took approximately 15 minutes and the subjects remained in a supine position during the scanning, wearing light clothing while lying flat on their back with arms by the side, without moving during the measurement. The results were transmitted to a connected computer for further analysis, according to the manufacturer orientation.

All Dual-energy X-ray absorptiometry- DXA measurements were made at the laboratory of the São Paulo State University Campus of Presidente Prudente, in a room with controlled temperature. Each morning before beginning the measurements, the equipment was calibrated by the same researcher and, according to the references provided by the manufacturer.

### Pubertal stages

Pubertal stage was self-assessed by the participants aged older than 10 years. A standardized series of drawings was given to the subjects to assess their own pubertal development according to Tanner, *et al.* (1969, 1970) [11,12]. Girls received drawings with the five pubertal stages of Tanner with breast and female pubic hair development, with appropriate descriptions. Boys received drawings with the five pubertal stages of Tanner with male genitalia and pubic hair development, with appropriate descriptions. It was asked to the subjects to select the drawing stage that best indicated his/her own development. The results were placed in a locked box by each subject to guarantee the integrity and anonymity of the subjects. The five stages of Tanner of pubic hair development were grouped in three classifications: Group 1 = subjects aged under 10 years and those who answered stage 1; Group 2 = subjects who answered stages 2 and 3; Group 3 = Subjects who answered stages 4 and 5.

### Blood samples

After a 12 hour fasting, blood samples were collected, by nurses, on tubes containing EDTA, an anticoagulant for blood samples. All blood samples were analysed in a private laboratory. Triglycerides, total cholesterol (TC), high-density lipoprotein cholesterol (HDL-C), low-density lipoprotein cholesterol (LDL-C), insulin and glucose were analysed, using the colorimetric method (dyslipidemia was identified as any modification in the lipid variables). The dosage of insulin was performed using the Elisa Kit (RayBio® Human Insulin ELISA Kit), according to the manufacturer instructions.

### Blood pressure

Systolic blood pressure (SBP) and diastolic blood pressure (DBP) values were measured with an electronic device (MX3Plus; Omron Corporation, Kyoto, Kansai, Japan), previously validated for pediatric populations [13]. The measurements were taken after 15 minutes of rest in a sitting position. Two measures were taken on the right arm, with a 2-minute interval between them and mean value of both measures was used. Two types of cuffs were used according to arm circumference. The classification of elevated blood pressure (EBP) was made by 95^th^ centile or higher for age, gender and height, according to National High Blood Pressure Education Program Working Group on High Blood Pressure in Children and Adolescents (NHBPEP, 2004) [14].

### Metabolic syndrome

The presence of MS was identified according to the cut offs proposed by World Health Organization adapted for children and adolescents [15]. The diagnosis of MS was based in the presence of, at least, three criteria. 1) Obesity: BMI ≥ 95th centile; 2) Glucose homeostasis: defined by hyperinsulinemia according to the norms for pubertal stage: pre-pubertal ≥15 mU/L, mid-puberty (stages 2–3) ≥ 30 mU/L [16], and post-pubertal ≥20 mU/L (adult value) and/or fasting glucose ≥ 110 mg/dL; 3) Hypertension: systolic blood pressure (SBP) ≥ 95th centile for age, gender and height [14]; 4) Dyslipidaemia: triglycerides >155 mg/dL, high density lipoprotein (HDL) < 35 mg/dL and total cholesterol ≥ 95th centile. Homeostasis model assessment-insulin resistance (HOMA-IR) was calculated according to the formula proposed by Matthews *et al*. (1985) [17]: HOMA-IR = Fasting insulin (mU/mL) X fasting glucose (mgl/dL)/405.

### Ultrasound

An ultrasound equipment (Toshiba Aplio Model Tochigi-ken, Japan) was used to assess the fat accumulation and the morphology of the liver (size of the left and right lobes), by a specialist medical doctor. NAFLD was stratified as followed: absent (0), mild (level 1), moderate (level 2) and severe (level 3). However, the sample was dichotomized: Absence and Presence, because of the lack of subjects with severe level. The ultrasound also measured intra-abdominal (IAAT) and subcutaneous (SCAT) abdominal adipose tissue thickness (cm). The values of IAAT and SCAT were stratified into quartile (Quartile_1_; Quartile_2-3_; Quartile_4_).

### Statistical analysis

Numerical variables were presented as mean and standard deviation. Test-T was used for comparison of the general variables between sexes. Chi-square test compared the categorical variables. Binary logistic regression (odds ratio [OR] and 95% confidence interval [OR_95%CI_]) indicated the magnitude of the associations adjusted by potential confounders (sex, age, maturation, classification of NAFLD and HOMA-IR). Statistical significance was set at 5% and the Statistical Package for Social Science, version 17.0 (SPSS Inc, Chicago, Illinois) was used for all analyses.

## Results

The comparison between male and female subjects (Table 1), showed similarity for age (p = 0.994), all biochemical variables, including, insulin resistance (p = 0.356) and dyslipidemia (p = 0.391) and blood pressure at rest (SBP with p = 0.426 and DBP with p = 0.551) and elevated (p = 0.740). The prevalence of MS was high in both genders (male 52.3% and female: 42.6%; p = 0.381). Boys presented lower percentage of body fatness (p = 0.012), however, higher IAAT than girls (p = 0.001).

**Table 1:** General characteristics of obese youth stratified by sex (n = 182)

	**Total (n = 182)**	**Male (n = 88)**	**Female (n = 94)**	**P**
	Mean (SD)	Mean (SD)	Mean (SD)	
Age (years)	11.0 (2.7)	11.1 (2.8)	11.1 (2.7)	0.994
Fasting glucose (mg/dL)	82.0 (7.1)	82.7 (7.2)	81.1 (6.9)	0.126
Triglycerides (mg/dL)	115.11 (51.3)	117.7 (58.5)	112.6 (43.7)	0.507
Cholesterol (mg/dL)	163.5 (32.9)	164.8 (36.3)	162.3 (29.4)	0.601
HDL-C (mg/dL)	43.1 (9.9)	42.9 (10.4)	43.3 (9.4)	0.771
LDL-C (mg/dL)	97.4 (28.9)	98.4 (31.8)	96.4 (25.9)	0.641
IAAT (cm)	4.4 (1.6)	4.81 (1.65)	3.97 (1.34)	0.001
SCAT (cm)	3.3 (1.4)	3.34 (1.55)	3.22 (1.28)	0.550
%BF	46.1 (5.4)	44.6 (5.5)	46.6 (4.8)	0.012
TrunkBF (kg)	17.9 (6.9)	15.2 (7.1)	14.3 (7.8)	0.440
SBP (mmHg)	116.3 (19.6)	117.5 (22.4)	115.2 (16.5)	0.426
DBP (mmHg)	69.0 (11.9)	68.4 (13.4)	69.5 (10.2)	0.551
NAFLD (%)	26.4	36,4	17.0	0.005
MS components (%)				
Hyperinsulinemia	65.4	69.3	61.7	0.356
EBP	35.7	37.5	34	0.740
Dyslipidemia	46.2	50	42.6	0.391
MS	48.4	52.3	44.7	0.381

*SD* standard-deviation, *HDL-C* high density lipoprotein cholesterol, *LDL-C* low density lipoprotein cholesterol, *SBP* systolic blood pressure; *DBP* diastolic blood pressure, *IAAT* intra-abdominal adipose tissue, *SCAT* subcutaneous adipose tissue, *%BF* percentage of body fatness, *EBP* elevated blood pressure, *MS* metabolic syndrome, *NAFLD* non-alcoholic fat liver disease.

Higher quartile of SCAT was associated with EBP (chi-square with p = 0.015 (Table 2), but not with NAFLD (p = 0.665). However, IAAT was positively associated with increased occurrence of dyslipidemia (p = 0.001), MS (p = 0.013) and NAFLD (p = 0.005). Hyperinsulinemia was not associated with SCAT and IAAT. The classification of NAFLD was positively associated with insulin resistance (Table 3), and marginally associated with MS (p = 0.075).

**Table 2:** Association between abdominal adipose tissue, non-alcoholic fatty liver disease and metabolic syndrome in obese youth (n = 182)

	**Subcutaneous abdominal adipose tissue (Quartile)**			**P**
**MS components**	**<P25**	**P25-P75**	**≥P75**	
Hyperinsulinemia	66.7%	63.3%	68%	0.866
EBP	28.6%	30%	52%	0.015
Dyslipidemia	50%	51.1%	34%	0.108
MS	45.2%	45.6%	56%	0.285
NAFLD	21.4%	28.9%	26%	0.665
	Intra-abdominal adipose tissue (Quartile)			P
MS components	<P25	P25-P75	≥P75	
Hyperinsulinemia	58.5%	66.3%	69.6%	0.287
EBP	31.7%	32.6%	45.7%	0.165
Dyslipidemia	22%	49.5%	60.9%	0.001
MS	34.1%	48.4%	60.9%	0.013
NAFLD	14.6%	24.2%	41.3%	0.005

*P*25 25^th^ percentile; *P*75 = 75^th^ percentile, *EBP* elevated blood pressure, *MS* metabolic syndrome, *NAFLD* non-alcoholic fat liver disease.

**Table 3:** Association between non-alcoholic fatty liver disease and metabolic syndrome components in obese children and adolescents (n = 182)

	**Non-alcoholic fatty liver disease**		**P**
**MS components**	**No**	**Yes**	
Hyperinsulinemia	60.4%	79.2%	0.031
EBP	35.1%	37.5%	0.900
Dyslipidemia	42.5%	56.3%	0.143
MS	44%	60.4%	0.075

*EBP* elevated blood pressure, *MS* metabolic syndrome.

In the multivariate model, statistically significant associations in the chi-square test were analyzed under adjustment of potential confounders (sex, age, maturation, presence of NAFLD and HOMA-IR). The association between higher quartile of SCAT and EBP did not reach statistical significance, in both adjusted models (Model 1- OR = 1.86 [0.70 – 4.91]; p = 0.210; Model 2- OR = 1.90 [0.71 – 5.05]; p = 0.195). On the other hand, both intermediate (OR = 3.33 [1.39 – 8.00]; p = 0.007) and highest (OR = 5.23 [1.92 – 14.2]; p = 0.001) quartile of IAAT were significantly associated to dyslipidemia, independently of age, sex, maturation, NAFLD and HOMA-IR (Table 4).

**Table 4:** Multivariate model for association between abdominal adipose tissue and metabolic syndrome components (n = 182)

**Abdominal adipose tissue**		**Binary logistic regression**		**Binary logistic regression**		**Binary logistic regression**	
		**Model – 1**		**Model – 2**		**Model – 3**	
	SCAT	OR (OR_95%CI_)	p	OR (OR_95%CI_)	p	OR (OR_95%CI_)	p
EBP	<P25	1.00	---	1.00	---	1.00	---
	P25-75	1.07 (0.47 – 2.41)	0.867	0.89 (0.38 – 2.07)	0.795	0.88 (0.38 – 2.05)	0.776
	≥P75	2.71 (1.13 – 6.46)	0.025	1.86 (0.70 – 4.91)	0.210	1.90 (0.71 – 5.05)	0.195
	IAAT	OR (OR_95%CI_)	p	OR (OR_95%CI_)	p	OR (OR_95%CI_)	p
Dyslipidemia	<P25	1.00	---	1.00	---	1.00	---
	P25-75	3.48 (1.50 – 8.07)	0.004	3.48 (1.46 – 8.31)	0.005	3.33 (1.39 – 8.00)	0.007
	≥P75	5.53 (2.14 – 14.2)	0.001	5.65 (2.09 – 15.2)	0.001	5.23 (1.92 – 14.2)	0.001

Model-1: crude analysis; Model-2: analysis adjusted by sex, age and maturation; Model-3: analysis adjusted by sex, age, maturation, NAFLD and HOMA-IR; *EBP* elevated blood pressure, *NAFLD* non-alcoholic fat liver disease, *OR* odds ratio, *95% CI* 95% confidence interval, *IAAT* intra-abdominal adipose tissue, *SCAT* subcutaneous adipose tissue, *EBP* elevated blood pressure, *MS* metabolic syndrome.

## Discussion

Despite seeming higher in obese youth, MS prevalence is not clear, because there is not yet a standard reference table worldwide accepted [18]. In our obese sample, boys (52.3%) and girls (44.7%) presented higher prevalence of the outcome than in another study with obese Brazilian adolescents (27,6%) [19] that used the same classification for MS. Moreover, the prevalence of NAFLD in our obese sample (26.4%) was similar to those recently observed in other overweight/obese adolescents (27,7%) [20]. Therefore, MS and NAFLD should be a relevant concern in developing countries, which experienced rapid industrialization process during the last decades, but without a consistent health politics related to nutrition and promotion of physical activity.

NAFLD was significantly associated with increased fasting insulin and glucose and marginally with MS. Previously, Ayonrinde *et al*. (2011) [21] identified in 1170 Australian adolescents that fat accumulation in the liver is related to decreased values of insulin. Other components of MS presented similar result of association with NAFLD, which is in accordance with previous studies, that refers to NAFLD as the “hepatic manifestation” of the MS [22].

Regarding the association between IAAT and dyslipidemia, the scientific literature has offered support to the idea that insulin resistance plays key role in the genesis of the NAFLD and components of MS [4], mainly in cases when the abdominal obesity is present. Adipose tissue has an endocrine function that produces multiple adipokines causing inflammatory process and insulin resistance [4,23,24]. Visceral adipose tissue located at abdominal region has particularities related to higher lipolysis, higher release of adipokines and these characteristics increase its atherosclerotic capacity [4].

Adipokines like tumor necrosis factor-α (TNF-α) and interleukine-6 are largely produced by abdominal fat and have been related to insulin resistance. TNF-α interferes on insulin metabolism cascade, activating proinflammatory pathways that impair insulin signalling at the level of the insulin receptor substrate proteins, causing insulin resistance [25]. Besides, TNF-α inhibits adiponectin expression, causes elevation in serum free fat acid by stimulation of lipolysis and hepatic lipogenesis, contributing for insulin resistance and may lead to development of NAFLD [4,23,26], justifying our findings.

Our results indicated that IAAT was more important than SCAT in the genesis of the MS and NAFLD [27]. Although SCAT has been associated with blood pressure and, in fact, the above mentioned pathways are strongly related to endothelial dysfunction [4], the significance was lost after the adjustments. Various studies have shown that, the increase of free fat acid in circulation corroborates with formation of low density lipoproteins (LDL) e very low density lipoproteins (VLDL) associated to reduction on high density lipoproteins (HDL) and very high density lipoproteins (VHDL), which results in atherosclerosis and elevation of arterial pressure [28,29].

Limitations of the study should be highlighted. The cross-sectional design constitutes a limitation because it is not possible to establish a causality of the involved variables. Moreover, the absence of pro-inflammatory adipokines analysis should be considered in the interpretation of the findings. In addition, the NAFLD was not diagnosed by liver biopsy, which is considered the gold standard, otherwise, the ultrasound is considered a useful tool for initial screening [30,31], based on parameters such as hepatorenal echo contrast and liver echogenicity [32]. Another reason is the unclear perform of serum aminotransferases, such as ALT and AST to diagnosis NAFLD in adults and children [33].

Furthermore, interventional studies are necessary to offer consistent support to our proposed model of analysis.

## Conclusions

In summary, our findings identified that, in obese adolescents, intra-abdominal fat seems to be more relevant to the development of MS and NAFLD than subcutaneous fat, and these results are independent of possible confounding variables. However, future studies with larger sample are necessary to confirm this evidence and perhaps, suggest a cut off of visceral fat to predict MS and NAFLD.

## Abbreviations

NAFLD: Nonalcohoolic fatty liver disease; MS: Metabolic syndrome; IAAT: Intra-abdominal adipose tissue; SCAT: Subcutaneous adipose tissue; BMI: Body mass index; HOMA-IR: Homeostasis model assessment-insulin resistance; BW: Body weight; %BF: Percentage of body fat; TFM: Trunk fat mass; TC: Total cholesterol; HDL-C: High-density lipoprotein cholesterol; LDL-C: Low density lipoproteins; SBP: Systolic blood pressure; DBP: Diastolic blood pressure; EBP: Elevated blood pressure; TNF-α: Tumor necrosis factor-α; VLDL: Very low density lipoproteins.

## Competing interests

The authors declare no competing interests.

## Authors’ contributions

LSS participated in the design of the study, was the main responsible for collection, analysis and interpretation of data, and also drafting the manuscript. PAM and BMMA carried out the Dual energy X-ray absorptiometry involved in analysis and interpretation of data and drafted the manuscript. PMS carried out the immunoassays and also in critical revision of the paper. RAF participated in the design of the study and performed the statistical analysis. DGDC participated in the design of the study and review the manuscript. IFFJ conceived of the study and involved in revising the manuscript critically for important intellectual content. All authors read and approved the final manuscript.

## Pre-publication history

The pre-publication history for this paper can be accessed here:

http://www.biomedcentral.com/1471-2431/13/115/prepub
